# Isolation and Identification of Endophytic *Chaetomium* sp. Strain V3 from *Ambrosia* and Its Effects on Tomato Plant Growth

**DOI:** 10.3390/jof11120870

**Published:** 2025-12-07

**Authors:** Yuping Jiang, Nazish Mehnaz, Bing Song, Mengyu Sun, Leibei Yang, Xuemei Li, Yueying Li, Lanlan Wang, Ze Wang, Yuzhu Dong, Lianju Ma

**Affiliations:** 1 College of Life Science, Shenyang Normal University, Shenyang 110034, China; 18341576116@163.com (Y.J.); 15041378687@163.com (B.S.); 18840538636@163.com (M.S.); 15940069617@163.com (L.Y.); lxmls132@163.com (X.L.); yueyinglicn@163.com (Y.L.); wangqi5387402006@163.com (L.W.); aca_zq@163.com (Z.W.); 2 Department of Microbiology and Molecular Genetics, The Women University Multan, Multan 60000, Pakistan; nazishmehnaz74@gmail.com

**Keywords:** Ragweed, endophytic fungi, *Chaetomium* sp., growth-promoting mechanisms, phytohormones, ARF

## Abstract

Ragweed (*Ambrosia artemisiifolia* L.), an invasive species, is well-known for its rapid growth, strong reproductive potential, and high stress tolerance. The evolutionary distinctiveness and strong ecological adaptability of *Ambrosia* have enabled the endophytic fungi that coevolved with it to become valuable microbial resources. In this study, one of the endophytic fungi isolated from ragweed was named strain V3. Strain V3 was identified as *Chaetomium* sp. (*Ascomycota*) based on morphological characteristics and molecular analyses. The strain V3 promotes tomato growth by significantly increasing plant height, root length, the number of lateral roots, and chlorophyll content, effectively enhancing photosynthesis and consequently improving fruit yield. Meanwhile, compared to the control, tomato fruits inoculated with strain V3 exhibited significantly higher levels of vitamin C (VC) and lycopene, indicating a notable enhancement in fruit quality. Additionally, strain V3 is capable of producing phytohormones, including indole-3-acetic acid (IAA), gibberellin (GA_3_), and zeatin, and of regulating the expression of tomato auxin response factor (ARF) genes. This study demonstrates that strain V3 has the potential to promote tomato plant growth.

## 1. Introduction

Plant endophytic fungi are defined as fungal groups that colonize the tissues of healthy plants, where they reside for certain or all stages of their life cycle without inducing noticeable disease symptoms [1]. By establishing complex symbiotic relationships with their host plants, these microorganisms play crucial roles in promoting plant growth, enhancing stress resistance [2], and regulating secondary metabolism [3]. Studies have demonstrated that endophytic fungi can enhance the nutritional status of host plants by secreting phytohormones such as indole-3-acetic acid (IAA) and gibberellic acid (GA_3_) [4], solubilizing essential minerals including phosphorus and potassium, and producing hydrolytic enzymes such as protease and cellulase [5]. Additionally, they improve plant tolerance to both biotic and abiotic stresses by inducing systemic resistance [6].

In the field of invasion biology, the rampant spread of *Ambrosia artemisiifolia* L. (commonly known as giant ragweed), a globally recognized noxious weed, has persistently posed significant challenges to ecological conservation and agricultural production [7]. Previous studies have demonstrated that the endophytic fungal community in ragweed roots exhibits pronounced host specificity, and certain strains can enhance host growth by regulating soil nutrient cycling [8]. However, systematic research on the growth-promoting effects of ragweed endophytic fungi on non-host plants, as well as their underlying molecular mechanisms, remains limited [9]. Exploring ragweed endophytic fungal resources and elucidating their growth-promoting mechanisms not only provides new insights into the ecological adaptability of invasive plants but also offers potential strain resources for the development of agricultural biological agents [10].

*Chaetomium*, a globally distributed genus of ascomycetous fungi, demonstrates dual roles in promoting plant growth and enhancing disease resistance. Evidence suggests that *Chaetomium* species produce phytohormones such as IAA, which stimulate root development and enhance nutrient uptake efficiency in plants. Furthermore, studies have shown that chaetocin, a secondary metabolite produced by *Chaetomium globosum*, can play a role in managing the Fusarium–root-knot nematode disease complex in tomatoes through the induction of disease resistance [11]. A representative study on stress tolerance enhancement reported that the *Chaetomium cupreum* isolate T31 colonizes the rhizosphere of *Triticum aestivum* (wheat), alleviates salt stress, and promotes plant growth, resulting in an 18% increase in grain yield and a 23% enhancement in leaf chlorophyll content under 150 mM NaCl stress [12]. Notably, the plant endophytic fungus *Chaetomium globosum* DX-THS3 demonstrates significant potential for biomass conversion: its lignocellulolytic enzyme cocktail, optimized through solid-state fermentation, achieves activities of 237.04 U/g CMCase, 54.8 U/g FPase, and 987.33 U/g xylanase, with thermal stability over the range of 30–60 °C and tolerance to acidic conditions (pH 3–6) [13]. However, research on its plant growth-promoting effects remains limited, particularly in the context of soil-grown tomatoes. The underlying mechanisms, key influencing factors, and the interaction process with the root system are still not well understood, which hinders their targeted and effective application. To address this knowledge gap, the present study conducted experiments using soil-grown tomatoes as the experimental system, aiming to elucidate the unclear growth-promoting mechanisms of *Chaetomium* in soil-based cultivation and to provide empirical data to support its development as a beneficial microbial resource.

Tomato (*Solanum lycopersicum* L.) is a globally important vegetable crop, with its yield and quality being significantly influenced by soil environment, nutrient availability, and hormonal regulation [14]. Among the key quality parameters, vitamin C (VC) and lycopene serve as primary indicators of nutritional value, whereas nitrate nitrogen and organic acids are directly associated with edible safety and flavor attributes. Precise modulation of these components represents a central objective in tomato quality improvement research. As a key component of biological fertilizers, endophytic fungi have garnered increasing attention due to their environmentally sustainable nature and consistent efficacy in promoting plant growth and enhancing quality traits [15]. Research has demonstrated that auxin response factors (ARFs) are essential for multiple stages of tomato growth and development [16], with the regulation of ARF gene expression being critical from seed germination to fruit ripening [17]. Therefore, investigating the effects of *Chaetomium* on ARF gene expression in tomatoes will facilitate the revelation of molecular mechanisms underlying the interactions between endophytic fungi and plants, thereby providing a theoretical basis for the precise regulation of plant growth [18].

Building on the aforementioned research background, this study systematically investigated the growth-promoting mechanisms of strain V3 and its effects on tomato growth through a comprehensive approach involving enzyme activity assays, pot experiments, field trials, high-performance liquid chromatography (HPLC), and real-time quantitative polymerase chain reaction (qRT-PCR) analysis.

## 2. Materials and Methods

### 2.1. Isolation and Identification of Endophytic Fungus

#### 2.1.1. Sample Processing and Isolation

Three healthy *Ambrosia artemisiifolia* L. plants were collected from the experimental field at Shenyang Normal University (41.908781° N, 123.41526° E), Shenyang, Liaoning Province, China. The sampling location was specifically chosen to ensure a diverse range of endophytic fungi due to the plant’s natural growth conditions in this region. Surface sterilization of plant roots, stems, and leaves was performed by immersion in 75% ethanol for 2 min, followed by treatment with 0.1% sodium hypochlorite for 1 min. Subsequently, the samples were rinsed three times with sterile distilled water to ensure complete removal of residual disinfecting agents. Tissue segments (approximately 0.5 cm in length) excised from roots, stems, and leaves were inoculated onto potato dextrose agar (PDA) medium supplemented with chloramphenicol and incubated under sterile conditions at 25 °C for 7 to 10 days. As a blank control, 1 mL of sterile distilled water was evenly spread on the PDA medium (Each organization conducted 3 repetitions, resulting in a total sample size of 27). Fungal colonies exhibiting distinct morphological characteristics were isolated and purified using the apical purification method from contamination-free experimental groups (as confirmed by sterile control groups) [19]. The ten isolated strains were designated as V1–V10 and preserved on PDA slants at 4 °C for subsequent analysis. (All the drugs and reagents used in this article are provided by Shanghai Sangon Biotech, Shanghai, China).

#### 2.1.2. Morphological Identification

The purified endophytic fungus was inoculated onto PDA plates and cultured at 25 °C until complete colony formation was observed. Colony morphological characteristics, including size, color, texture, and margin shape, were systematically recorded. Hyphal and spore morphologies were examined under an optical microscope at a magnification of 400×. Morphological identification was conducted by comparing the observed characteristics with those described in the Manual of Fungal Identification [20].

#### 2.1.3. Molecular Biological Identification

The purified endophytic fungus was inoculated into potato dextrose broth (PDB) liquid medium and cultured with constant shaking (130 rpm) at 25 °C for 7 d until sufficient mycelial growth was observed. Fungal genomic DNA was extracted using a rapid fungal genomic DNA extraction kit (Shanghai Sangon Biotech, Shanghai, China) according to the manufacturer’s instructions [21]. PCR amplification was performed using the ITS1 and ITS4 primers (ITS1: 5′-TCCGTAGGTGAACCTGCGG-3′ and ITS4: 5′-TCCTCCGCTTATTGATATGC-3′ for amplifying the ITS region). The conditions were as follows: initial denaturation at 95 °C for 5 min, followed by 35 cycles of denaturation at 95 °C for 30 s, annealing at 55 °C for 30 s, extension at 72 °C for 45 s, and a final extension at 72 °C for 10 min. Amplification products were detected via 1% agarose gel electrophoresis stained with GelRed (Biotium, Fremont, CA, USA). Samples were sent to Shanghai Sangon Biotech Co., Ltd. for DNA sequencing. Homology analysis of the sequences was conducted using BLAST “https://blast.ncbi.nlm.nih.gov/Blast.cgi” (accessed on 8 October 2025), and a phylogenetic tree was constructed based on the neighbor-joining method using MEGA 11.0.

### 2.2. Determination of Growth-Promoting Characteristics

#### 2.2.1. Hydrolytic Enzyme Activity Assay

- Protease activity: Strain V3 was inoculated onto skim milk medium (containing 1% skim milk) and cultured in the dark at 25 °C for 7 d. The presence of transparent zones around the colonies was observed as an indicator of protease activity [22].
- Cellulase activity: Carboxymethyl cellulose sodium (CMC-Na) was used as the sole carbon source. After culturing in the dark at 25 °C for 7 d, the medium was stained with 1 g/L Congo red solution for 2 h. The staining solution was then discarded, and the medium was soaked in a 1 M NaCl solution for 3 h to decolorize. Transparent zones around the colonies were observed as an indicator of cellulase activity.

#### 2.2.2. Phytohormone Determination

Strain V3 was inoculated into PDB medium and cultured with shaking (120 rpm) at 25 °C for 30 d to ensure the full production of secondary metabolites. The fermented culture was filtered, and 1.5 L of the fermentation broth was extracted twice with an equal volume of ethyl acetate (each extraction lasting 3 h). The supernatant extract was subjected to rotary evaporation at 30 °C in the dark until the volume reached a constant level. The residue was dissolved in 2 mL of 40% methanol solution (containing trace amounts of glacial acetic acid) and prepared in 3 replicates. The solution was then filtered through a 0.22 μm membrane filter to obtain the final filtrate.

The concentrations of IAA, GA_3_, and zeatin in the fermentation broth were quantified using HPLC [23]. Chromatographic conditions: detection wavelength 210 nm, mobile phase consisting of methanol-water binary solvent (2:3 ratio) with 0.2% glacial acetic acid added to suppress solute ionization, flow rate 1 mL/min, and column temperature maintained at 30 °C.

Chromatographic analysis was performed using ultra-high performance liquid chromatography–tandem mass spectrometry (UPLC-MS/MS) on a Waters ACQUITY UPLC I-Class system coupled with a Xevo TQ-S micro triple quadrupole mass spectrometer (Waters Corporation, Milford, MA, USA). To ensure the accuracy and reliability of quantification, standard curves for IAA, GA_3_, and zeatin were established with a concentration range of 0.1 to 100 ng/mL, including calibration points at 0.1, 0.5, 1, 5, 10, 50, and 100 ng/mL. All calibration curves exhibited correlation coefficients (R^2^) exceeding 0.999, confirming excellent linearity for quantitative analysis. The limit of detection (LOD) was determined based on a signal-to-noise ratio (S/N) of 3:1. The initial experiment did not include a sterile potato dextrose broth (PDB) control group; therefore, the detection of phytohormones (IAA, GA_3_, and zeatin) in the fungal culture medium may be influenced by components of the medium itself, and their presence cannot be solely attributed to fungal production.

### 2.3. Pot and Field Experiments

#### 2.3.1. Pot Experiment

The strain V3 culture plate at the logarithmic spore-producing stage—cultivated in the dark at 28 °C for 7 d to ensure a spore yield of at least 1 × 10^7^ per plate—was used for spore collection. A sterile cotton swab was gently rubbed across the surface of the plate in a direction opposite to mycelial growth. Spores were transferred into a 50 mL centrifuge tube containing 15 mL of sterile 0.02% Tween-80 solution. Following vortexing at 2000 rpm for 2 min, the suspension was filtered through two layers of sterile gauze (pore size approximately 100 μm) to remove mycelial fragments and residual culture medium particles, and the filtrate was collected as the crude spore suspension. The 5% (*w*/*v*) fungal spore suspension used in this experiment had a final spore concentration of 5 × 10^6^ spores/mL, as determined by hemocytometer counting, with a germination rate of no less than 85%, satisfying both the quantitative and viability requirements for plant inoculation.

Hoagland nutrient solution: Among them, macronutrients include four compounds: 2.5 mmol/L Ca(NO_3_)_2_·4H_2_O; 2.0 mmol/L KNO_3_; 0.5 mmol/L KH_2_PO_4_; 1.0 mmol/L MgSO_4_·7H_2_O. Micronutrients include six compounds: 0.1 mmol/L Na_2_FeEDTA; 0.025 mmol/L H_3_BO_3_; 0.002 mmol/L MnSO_4_·H_2_O; 0.002 mmol/L ZnSO_4_·7H_2_O; 0.0005 mmol/L CuSO_4_·5H_2_O; 0.0001 mmol/L (NH_4_)_6_Mo_7_O_24_·4H_2_O [24].

Healthy tomato seeds (variety ‘Jinpeng No.1’) were soaked in 25 °C water for 4 h and germinated at 30 °C for 36 h. Germinated seeds were sown in plug trays with seedling soil and raised in a greenhouse (temperature: 25 ± 2 °C, humidity: 60–70%, light cycle: 16 h light/8 h dark) until the two-leaf-and-one-heart stage. Subsequently, seedlings were transferred individually into 500 mL cylindrical plastic cups, with one seedling per cup, and cultivated hydroponically using Hoagland’s nutrient solution. Each cup was supplied with 300 mL of the nutrient solution, which was replenished daily to maintain a constant volume. The nutrient solution was renewed every three days to ensure stable nutrient concentrations throughout the experiment. After 7 d of acclimatization, a 30 mL suspension containing 5% fungal inoculum was applied to tomato seedlings via the root-drenching method, whereas the control group was treated with an equal volume of PDB culture medium (three replicates per treatment). Following 28 d of continuous cultivation, plant height and root length were measured.

#### 2.3.2. Field Experiment

Seedlings were prepared and pre-cultured as described in the pot experiment, under the aforementioned hydroponic conditions, with growth assessments conducted 28 days post-inoculation. Subsequently, the seedlings were transplanted to experimental plots at Shenyang Normal University (32 m^2^ per plot, 32 plants per plot) and maintained under regular irrigation (500 mL/m^2^ every 3 days) and standardized field management practices, including a temperature range of 22–30 °C, natural photoperiod, and relative humidity of 55–75%. Plant height, number of lateral leaves, and lateral leaf length were recorded at biweekly intervals over three measurement cycles. At fruit maturity, leaf chlorophyll content, average fruit weight, VC content (determined by the 2,6-dichlorophenolindophenol method), lycopene content (measured via acetone extraction), and nitrate nitrogen content (quantified by ultraviolet spectrophotometry) were analyzed. The mature, fresh tomato pulp was homogenized into a uniform paste using a blender. The titratable acidity of the tomato fruit via acid-base titration was determined. The organic acid content was calculated according to the following formula: Organic acid content = (VNaOH × 0.1 × K × C)/(W × Vsample solution) × 100, where K = 0.67. Soluble carbohydrate concentration was determined using anthrone colorimetry with a detection wavelength of 620 nm [25]. C: Molar mass conversion factor for the titration reaction, used to convert the amount of consumed NaOH standard solution into the corresponding mass of organic acids. W: Sample weight (in g), representing the actual mass of the tomato sample used in the analysis. V: Volume of the sample extract utilized in titration (in mL), denoting the aliquot portion of the total extract subjected to NaOH titration

### 2.4. RNA Extraction and qRT-PCR

Tomato plants grown in the field for 1 month were sampled, and roots, stems, and leaves were collected. Based on existing literature, six ARF family genes were selected to analyze their expression patterns, thereby elucidating the molecular mechanism by which strain V3 promotes tomato plant growth [26]. Total RNA was extracted from these tissues according to the manufacturer’s instructions of the RNA extraction kit (Promega, Madison, WI, USA). Following the protocols provided in the TaKaRa Reverse Transcription PCR (RT-PCR) kit (Shanghai Sangon Biotech, Shanghai, China), the extracted total RNA was reverse-transcribed into single-stranded cDNA, which was subsequently stored at −20 °C for further analysis. For cDNA amplification, TaKaRa Taq (R001B) was utilized with the synthesized cDNA as the template. Quantitative real-time PCR (qRT-PCR) amplification was conducted using UBI3 as the reference gene (Table 1). The primer sequences for the PCR reactions were designed based on information retrieved from the NCBI database and relevant literature [27]. The calculation formulas are as follows: Ct (target gene) − Ct (reference gene) = ΔCt; ΔCt (treated sample) − ΔCt (control sample) = ΔΔCt; Fold change = 2^−ΔΔCt^ (where 2 represents the multiplicative change in amplicon quantity per cycle; ΔΔCt represents the corrected difference in cycle numbers between treated and control samples).

### 2.5. Root Colonization Observation

To verify successful colonization of tomato plants by strain V3 and the establishment of a symbiotic relationship, the roots were treated with 10% potassium hydroxide to remove cellular debris, acidified with 5% hydrochloric acid, and stained with aniline blue. Subsequently, hyphal colonization was examined under a fluorescence microscope.

### 2.6. Statistical Analysis

Data were analyzed via one-way ANOVA using SPSS 22.0 software, and differences between groups were compared using the LSD test (*p* < 0.05). Graphs were generated with Origin 2021 software, and correlation analysis was conducted using GraphPad Prism 8 software.

## 3. Results

### 3.1. Characterization of Chaetomium sp. V3: An Endophytic Fungus Isolated from Ragweed

**Figure 1:**
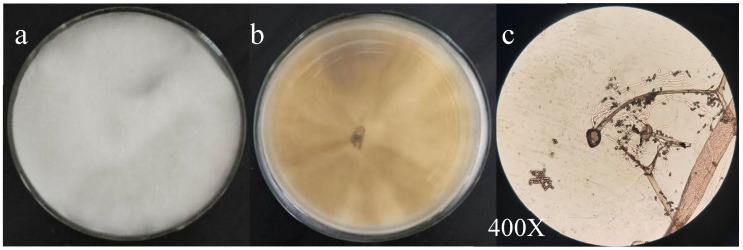
Colony morphology of *Chaetomium* sp. strain V3. (**a**) Front side of colony (cultured on PDA medium); (**b**) Back side of colony; (**c**) Microscopic observation of strain V3 (magnified 400 times).

Ten endophytic fungi were isolated from ragweed and named V1–V10. Promoting growth experiments related to tomato seeds were conducted, and the most effective strain, V3, was selected. Morphological and molecular biological identification was conducted. For strain V3: aerial hyphae were observed, exhibiting rapid growth; the hyphae were white and grew uniformly in an upright manner. The reverse side of the colony was gray with radial patterns. After 14 d of culture, a yellow oily liquid appeared on the surface of the hyphae, while the hyphae remained white. Microscopic examination revealed globose ascomata containing subglobose, deliquescent asci. The ascospores are lenticular, unicellular, and arranged in clusters (Figure 1c). Additionally, free spores exhibit a regular, round to oval morphology (Figure 1).

**Figure 2:**
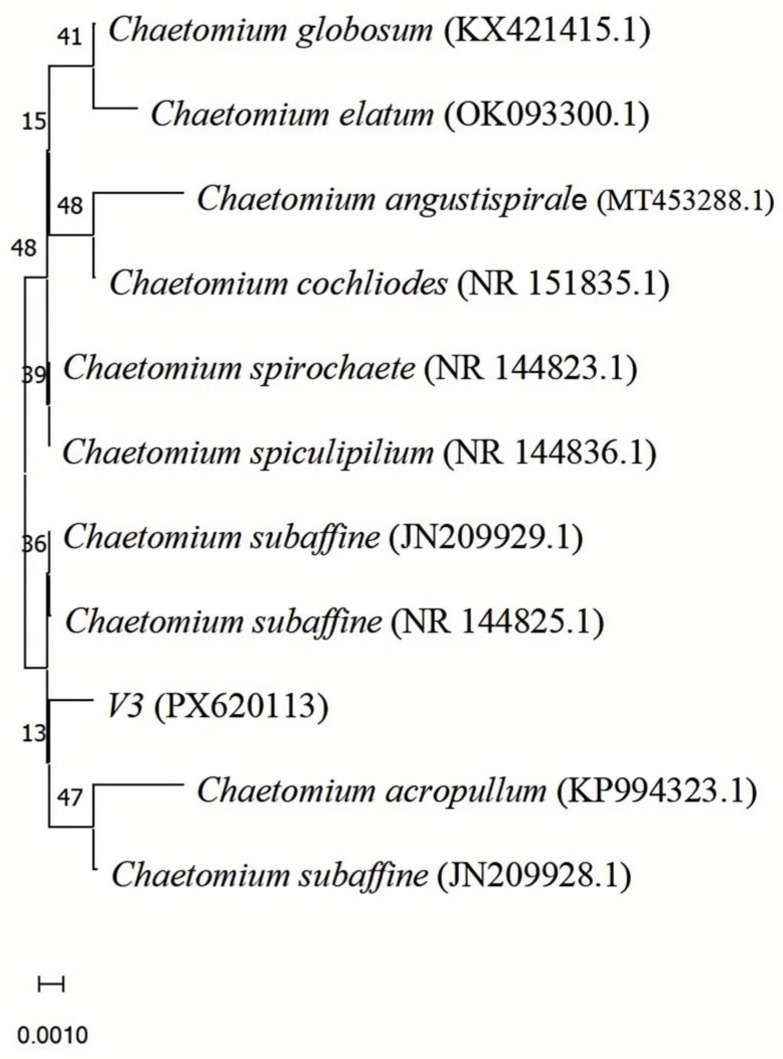
Phylogenetic tree based on the ITS sequences of strain V3 and 10 type strains of *Chaetomium* (retrieved from GenBank). The tree was inferred using the neighbor-joining method with 1000 bootstrap replicates; branch support values are indicated at the corresponding nodes.

Following morphological identification, the internal transcribed spacer (ITS) region of strain V3 was amplified and sequenced, and the resulting ITS sequence has been deposited in the GenBank database under accession number PX620113. Using this ITS sequence as a query, nucleotide sequence similarity searches were conducted in GenBank using the BLAST program. Representative sequences from reference strains—including *Chaetomium globosum* (KX421415.1), *Chaetomium elatum* (OK093300.1), *Chaetomium angustispirale* (MT453288.1), *Chaetomium cochliodes* (NR_151835.1), *Chaetomium spirochaete* (NR_144823.1), *Chaetomium spiculipilium* (NR_144836.1), *Chaetomium subaffine* (JN209929.1, NR_144825.1, JN209928.1), and *Chaetomium acropullum* (KP994323.1)—were retrieved from GenBank for phylogenetic analysis (Figure 2).

The initial BLAST analysis revealed that the ITS sequence of strain V3 exhibited the highest similarity (97.2%) to the type strain of *Chaetomium subaffine* CBS 637.91 (GenBank accession number: NR_144825). To further clarify its taxonomic position, additional BLAST searches were performed against the “Fungal Types” ITS database in GenBank, which contains authenticated type strains.

### 3.2. Analysis of Growth-Promoting Characteristics

#### 3.2.1. Hydrolytic Enzyme Activity

**Figure 3:**
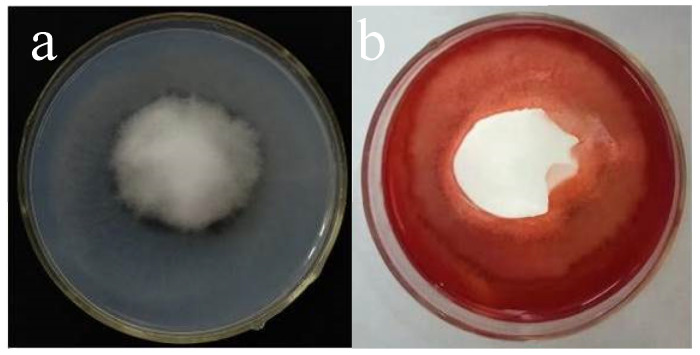
Determination of hydrolase activity produced by *Chaetomium* sp. strain V3. (**a**) Protease activity; (**b**) Cellulase activity.

The protease-producing medium used skim milk as the sole nitrogen source, and the medium was milky white in color. If the strain produced protease, a clear hydrolysis zone formed around the colony; otherwise, no clear zone appeared. As shown in Figure 3a, strain V3 exhibited protease-producing ability. The cellulase-producing medium was prepared with Congo red and used carboxymethyl cellulose (CMC) as the sole carbon source, with the medium appearing red in color. If the strain produced cellulase, a clear hydrolysis zone would form around the colony; otherwise, no clear zone would be observed. As shown in Figure 3b, strain V3 exhibited cellulase-producing ability.

#### 3.2.2. Plant Hormone Synthesis

**Figure 4:**
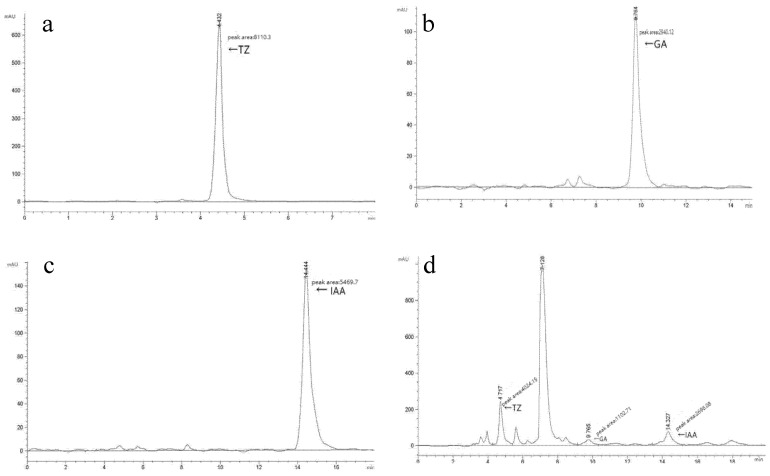
HPLC chromatograms for plant hormone content determination. (**a**) Zeatin standard; (**b**) GA_3_ standard; (**c**) IAA standard; (**d**) Fermentation broth of *Chaetomium* sp. strain V3 (cultured in potato-containing liquid medium).

The concentrations of IAA, GA_3_, and zeatin in the fermentation broth of strain V3 were quantified using HPLC. The zeatin standard exhibited a significant absorption peak at 4.432 s (Figure 4a), while the fermentation broth of strain V3 showed a prominent absorption peak at a retention time of 4.717 s (Figure 4a), with zeatin detected at a concentration of 0.2 ng/mL. The GA_3_ standard displayed a significant absorption peak at 9.764 s (Figure 4b), and the fermentation broth of strain V3 exhibited a smaller absorption peak at 9.765 s (Figure 4b), with GA_3_ detected at 0.014 ng/mL. The IAA standard presented a prominent absorption peak at 14.444 s (Figure 4c), and the fermentation broth of strain V3 also showed a small absorption peak at a retention time of 14.327 s (Figure 4d), with IAA detected at 0.071 ng/mL. However, due to the lack of a sterile PDB medium control (to exclude background hormone contributions from the medium itself), the origin of these detected phytohormones (whether produced by strain V3 or derived from the culture medium) remains to be clarified.

### 3.3. Chaetomium sp. Promotes the Growth of Tomatoes

**Figure 5:**
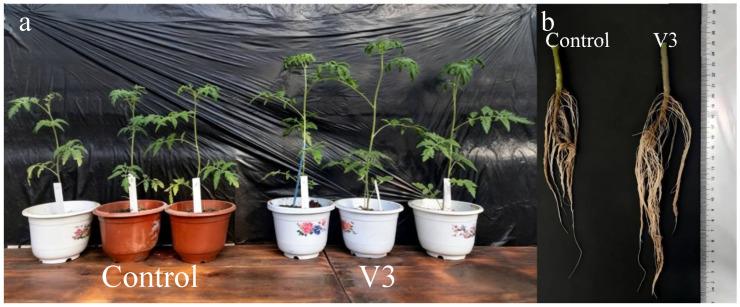
Effect of the endophytic fungus *Chaetomium* sp. strain V3 on the growth of tomato plants (*n* = 3). (**a**) Above-ground portions of tomato plants; (**b**) Root systems of tomato plants. Control: non-inoculated; V3: inoculated with *Chaetomium* sp. strain V3.

Strain V3 was applied to tomato seedlings in a potting growth-promotion experiment at a concentration of 5%. As shown in Figure 5, strain V3 significantly enhanced the growth of tomato plants, as evidenced by increases in plant height, leaf number, and root system development.

### 3.4. Field Growth and Quality Effects

#### 3.4.1. Growth and Yield Indicators

**Figure 6:**
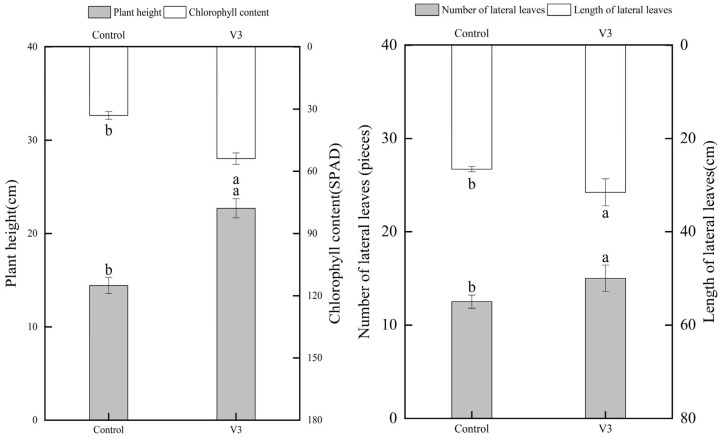
Effects of endophytic fungus *Chaetomium* sp. strain V3 on growth-related indicators of tomato plants (plant height, chlorophyll content, number of lateral leaves, length of lateral leaves). Different lowercase letters indicate significant differences among treatment groups (*n* = 16, *p* < 0.05).

One month after field transplantation, tomato seedlings inoculated with strain V3 were assessed (16 plants per treatment, with 3 technical replicates per plant), and growth-related indicators showed significant improvements: Plant height increased by 19% (mean: 23 cm, 95% CI (21–25), one-way ANOVA: F = 38.6, df = 30, *p* < 0.001), while chlorophyll content (expressed as SPAD value) rose by 49.03% (mean: 150, 95% CI (140–160), one-way ANOVA: F = 45.9, df = 30, *p* < 0.001)—suggesting enhanced photosynthetic capacity. Additionally, strain V3 promoted lateral shoot development: the number of lateral leaves increased by 28% (mean: 15 pieces, 95% CI (13–17), one-way ANOVA: F = 29.7, df = 30, *p* < 0.001), and lateral leaf length increased by 32% (mean: 60 cm, 95% CI (55–65), one-way ANOVA: F = 33.2, df = 30, *p* < 0.001). These phenotypic changes collectively improved the overall growth performance and photosynthetic efficiency of tomato plants (Figure 6).

#### 3.4.2. Fruit Quality Analysis

**Figure 7:**
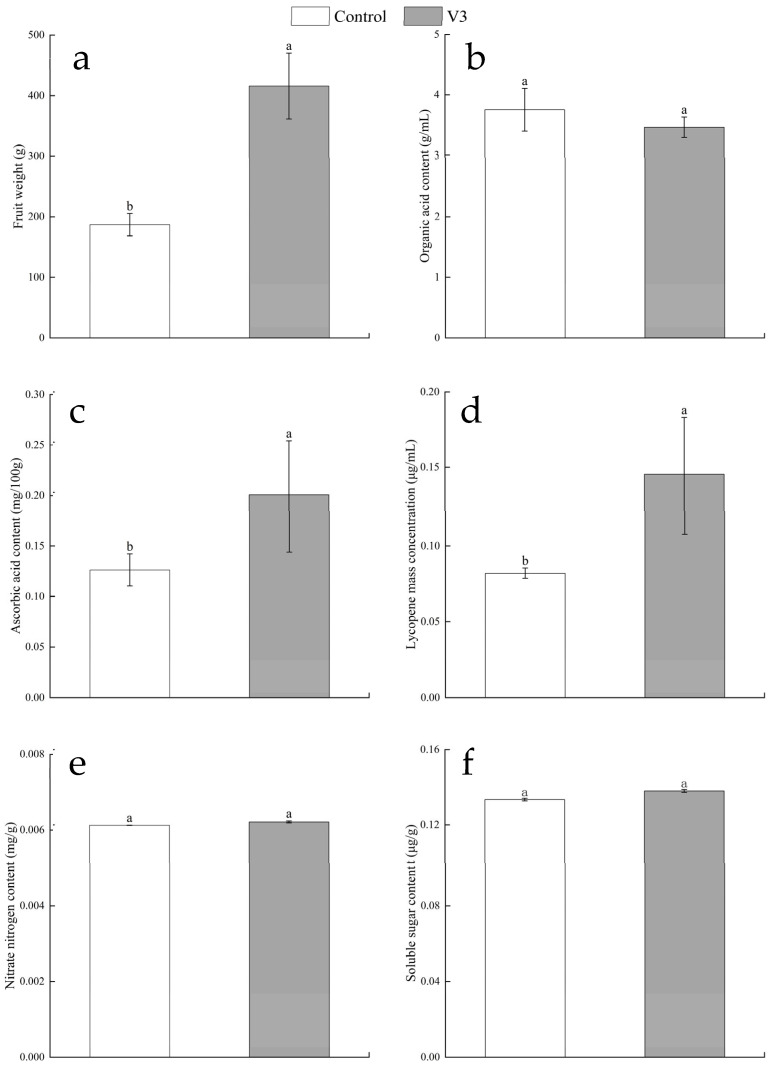
Effects of endophytic fungus *Chaetomium* sp. strain V3 on fruit yield and nutritional quality of tomato: (**a**) single fruit weight, (**b**) organic acid concentration, (**c**) ascorbic acid (VC) concentration, (**d**) lycopene concentration, (**e**) nitrate nitrogen concentration, (**f**) soluble carbohydrate (soluble sugar) concentration. Different lowercase letters indicate significant differences among treatment groups (*n* = 48, *p* < 0.05).

Three months after field transplantation, tomato fruits were harvested and their weights measured (Three mature fruits of uniform size and free from visible pests or diseases were collected from each tomato plant; for each treatment, 16 individual plants were selected, resulting in 48 fruits analyzed per group). The results showed that fruit weight in tomato plants treated with strain V3 increased significantly, reaching a maximum increase of 37.69% (mean: 410 g/fruit, 95% CI (360–460), one-way ANOVA: F = 42.8, df = 94, *p* < 0.001; Figure 7). Organic acid, VC, lycopene, and nitrate nitrogen contents in the fruits were also analyzed. The analysis revealed that organic acid content decreased slightly by 7.73% in strain V3-treated plants (mean: 3.5 g/mL, 95% CI (3.1–3.9), one-way ANOVA: F = 1.92, df = 94, *p* = 0.172), although this change was not statistically significant. In contrast, VC content increased by 56.44% (mean: 0.20 mg/100g, 95% CI (0.18–0.22), one-way ANOVA: F = 51.3, df = 94, *p* < 0.001), and lycopene content rose by 77.28% (mean: 0.15 mg/mL, 95% CI (0.13–0.17), one-way ANOVA: F = 67.5, df = 94, *p* < 0.001). Nitrate nitrogen content increased marginally by 1.45% (mean: 0.006 mg/g, 95% CI (0.005–0.007), one-way ANOVA: F = 0.35, df = 94, *p* = 0.556; Figure 7).

### 3.5. Analysis of ARF Gene Expression

**Figure 8:**
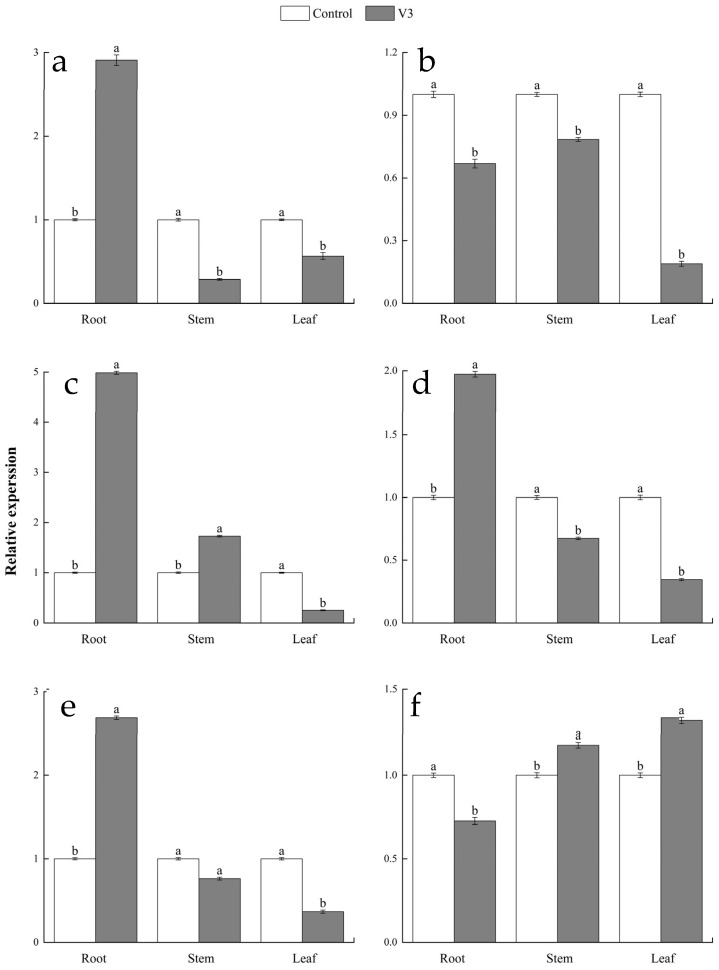
The effect of the endophytic fungus *Chaetomium* sp. strain V3 on the relative expression levels of six ARF family genes in the roots, stems, and leaves of tomato plants: (**a**) ARF2, (**b**) ARF4, (**c**) ARF7, (**d**) ARF8, (**e**) ARF10, (**f**) ARF12. Data are presented as the mean ± standard deviation (SD) from three independent experiments. Duncan’s multiple range test was used to analyze the statistical significance of differences between groups for all column charts, where columns marked with different letters indicate statistically significant differences (*n* = 3, *p* < 0.05).

An experiment was conducted to investigate the effect of strain V3 inoculation on the expression of six ARFs in tomato plants. As shown in Figure 8, following strain V3 treatment, the expression of ARF2, ARF7, ARF8, and ARF10 genes was upregulated in the roots, whereas the expression of ARF4 and ARF12 genes was downregulated. In the stems, the expression of ARF7 and ARF12 genes was upregulated, while other ARF genes were downregulated. In the leaves, only the ARF12 gene exhibited upregulation, with all other ARF genes being downregulated. Under identical treatment conditions, the expression profiles of these genes varied across different plant tissues, suggesting that they play distinct roles in promoting plant growth. Furthermore, their coordinated interactions regulate auxin levels, thereby influencing overall plant growth and development.

### 3.6. Observation of Root Colonization by Chaetomium sp. Strain V3

**Figure 9:**
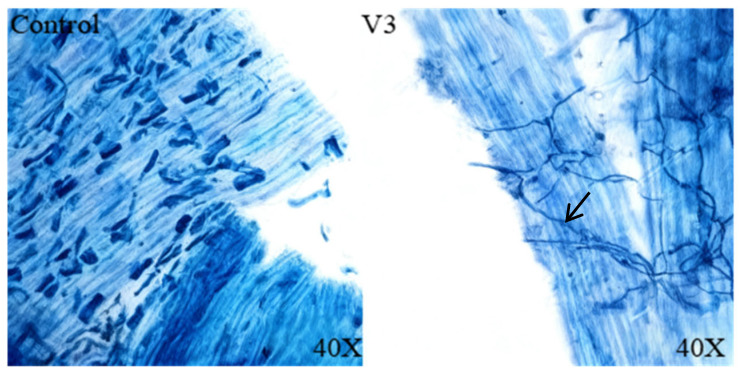
The colonization situation of the endophytic fungus *Chaetomium* sp. strain V3 in tomato roots (stained with phenylthiazole blue, magnified 40 times).

Two weeks after inoculation with the endophytic fungus, the tomato roots from both the experimental group and the control group were stained using trypan blue for observation. The results demonstrated that the colonization morphology of strain V3 in the tomato roots was clearly visible. Compared with the control group, circular spores and fungal hyphae structures were clearly observed within the root epidermal cells, as indicated by arrows (Figure 9). This observation confirms that strain V3 has successfully established a stable symbiotic relationship with the tomato roots.

## 4. Discussion

In this study, ten endophytic fungal strains were successfully isolated from ragweed, and strain V3 was identified as belonging to the genus *Chaetomium* sp. This strain exhibits multiple plant growth-promoting traits, contributing to enhanced tomato growth.

Soil proteins are degraded by proteases, resulting in the release of bioavailable nitrogen that can be directly absorbed and utilized by plants [28]. This process ensures a sustained supply of essential nitrogen nutrients necessary for tomato growth and mitigates growth limitations associated with inadequate soil nitrogen availability. Cellulose in soil is enzymatically degraded by cellulase [29]. This degradation process enhances soil aggregate stability, improves aeration, and increases water retention capacity, collectively creating favorable conditions for root development. These improved soil properties promote root elongation and branching while simultaneously enhancing the efficiency of water and nutrient uptake [30]. Through these interconnected mechanisms, tomato growth is facilitated by increased nutrient availability and optimized rhizosphere conditions. Experimental analysis in this study revealed that strain V3 exhibits both protease and cellulase activities (Figure 3). As demonstrated by Guillaume et al. [31], microbial enzymatic hydrolysis can improve plant growth conditions, indicating that the dual enzymatic activity of strain V3 may play a role in promoting plant growth.

From the perspective of hormone content, IAA, a canonical auxin, promotes cell elongation and division [32], GA_3_ alleviates seed dormancy [33], and zeatin primarily regulates cell division and differentiation [34], thereby ensuring coordinated development of tomato tissues and organs [35]. This demonstrated that IAA further stimulates root, stem, and leaf growth in tomatoes. Additional studies have shown that plant hormones not only promote seed germination and stem elongation but also participate in fruit development [36]. In this study, low concentrations of IAA (0.071 ng/mL), GA_3_ (0.014 ng/mL), and zeatin (0.2 ng/mL) were detected via HPLC in the liquid culture medium of Strain V3 (Figure 4). Although the origin of these hormones (whether produced by Strain V3 or derived from the medium itself) remains to be clarified due to the lack of a sterile medium control, their presence in the liquid culture system still suggests the potential of Strain V3 to modulate tomato growth (in line with the known physiological functions of these hormones). The physiological effects of these hormones are ultimately mediated through the differential expression of downstream genes. In this context, the modulation of tomato ARF gene expression by Strain V3 represents a key molecular mechanism underlying its regulatory role in plant growth.

An increase in chlorophyll content directly enhances the photosynthetic efficiency of tomatoes, thereby providing greater energy and organic nutrients to support plant growth [37]. Studies have shown that the promotion of tomato growth from seed germination through vegetative development is consistent with the growth-regulatory patterns commonly exhibited by beneficial endophytic fungi in their host plants [38]. In both pot experiments and field trials, strain V3 significantly improved seed germination rate, plant height, root length, and lateral root development (Figure 5), with chlorophyll content increasing by 49.03% (Figure 6). These findings further confirm the ability of strain V3 to promote tomato growth and enhance fruit quality.

VC exhibits antioxidant activity and enhances human immune function [39]. Lycopene effectively neutralizes free radicals in the human body [40]. In terms of yield, strain V3 significantly increased fruit weight and total tomato yield, with VC content rising by 56.44% (Figure 7c) and lycopene content increasing significantly by 77.28% (Figure 7d). Nitrate nitrogen is the primary form of bioavailable nitrogen in soil and is readily absorbed and utilized by plants. However, excessive accumulation of nitrate nitrogen in tomato fruits may lead to the formation of harmful compounds during human metabolism. The concentration and molar ratio of organic acids directly influence the acid-sweet balance of the fruit [41]. Regarding fruit quality, nitrate nitrogen levels under strain V3 treatment increased only slightly (Figure 7b), while organic acid content remained stable without significant reduction (Figure 7e). These results demonstrate that strain V3 not only enhances tomato yield but also improves nutritional value and flavor, achieving concurrent improvements in both yield and quality.

The ARF gene family, as a core component of the auxin signaling pathway, plays a pivotal role in determining how auxin regulates the growth and development of various plant tissues [42]. This study found that following treatment with strain V3, ARF genes in tomato plants exhibited significant tissue-specific expression patterns. In roots, the expression levels of ARF2, ARF7, ARF8, and ARF10 were markedly upregulated (Figure 8). Previous studies have demonstrated that ARF2 is involved in fine-tuning cell elongation during root development, and its upregulation enhances the longitudinal extension capacity of root cells [43]. ARF7 and ARF8 are key regulators of lateral root initiation; their synergistic action promotes the formation and emergence of lateral root primordia, thereby increasing lateral root density [35]. ARF10 is associated with root cap cell differentiation and nutrient perception, and its enhanced expression improves the root system’s efficiency in absorbing water and mineral elements from the soil [44]. The coordinated upregulation of these genes collectively promotes root system expansion and functional enhancement, establishing a stronger foundation for nutrient acquisition and overall plant growth.

In stem tissues, the expression of ARF7 and ARF12 was significantly elevated. Upregulation of ARF7 in stems promotes cell elongation and division at internodes, directly contributing to longitudinal growth and radial thickening [45]. ARF12 is linked to the maintenance of apical meristem activity in the stem, and its stable expression supports sustained shoot growth potential. The coordinated regulation of these genes results in stronger, more robust stems capable of effectively supporting leaves and fruits while improving the translocation of photosynthates and nutrients.

In leaf tissues, ARF12 expression was significantly upregulated. Although the precise role of ARF12 in leaves remains incompletely characterized, prior research suggests it may influence photosynthetic activity by regulating chloroplast development and the expression of photosynthesis-related genes [34]. The observed upregulation of ARF12 in this study may be directly linked to the increased chlorophyll content and improved photosynthetic efficiency in tomato leaves following application of the V3 strain. The underlying mechanism may involve activating chlorophyll biosynthesis genes (e.g., chlorophyll synthase genes), suppressing chlorophyll degradation pathways, or regulating proteins involved in photosystem II assembly, thereby enhancing light reaction efficiency. Collectively, these effects lead to greater accumulation of photosynthetic products, providing enhanced metabolic resources for plant growth.

ARF4 is a negative regulator of auxin response. An increase in its expression level will inhibit cell elongation, division, or organ differentiation [46]. The downregulation of these inhibitory ARF genes in roots, stems, and leaves is essentially due to V3, which reduces the “growth inhibitory signals” and thereby removes the constraints on the development of these organs—for example, the downregulation of inhibitory ARF genes in roots may promote root elongation, root hair formation, and enhance nutrient absorption.

**Figure 10:**
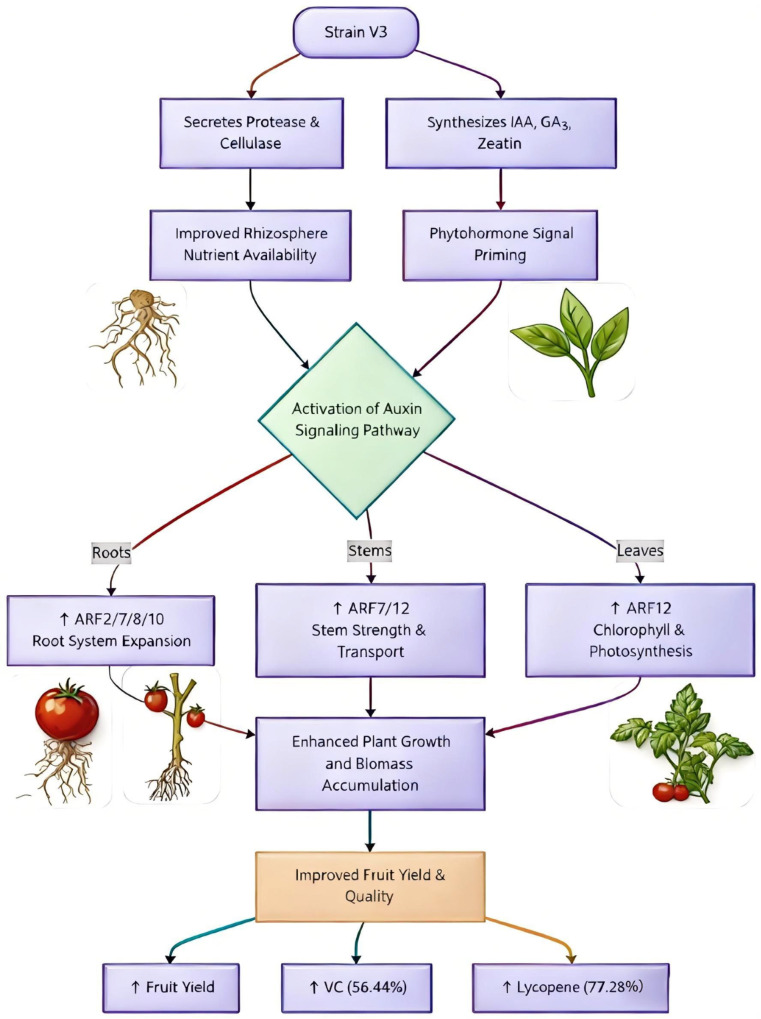
Conceptual model illustrating how the endophytic fungus *Chaetomium* sp. strain V3 modulates the tomato auxin signaling pathway to promote plant growth and enhance fruit quality. (↑: This arrow indicates an upward trend).

Strain V3 regulates tomato growth and quality through a dual synergistic mechanism: on one hand, it secretes proteases and cellulases to improve rhizosphere soil structure and enhance nutrient availability; on the other hand, it synthesizes IAA, GA_3_, and zeatin to activate the auxin signaling pathway by specifically modulating tissue-specific expression of ARF2, ARF7, ARF8, and ARF10 in roots and ARF12 in leaves. This regulatory network enhances root nutrient uptake capacity and leaf photosynthetic efficiency, with chlorophyll content increased by 49.03%. Ultimately, this leads to greater accumulation of photosynthetic products, resulting in higher fruit yield and significant improvements in VC (by 56.44%) and lycopene (by 77.28%) contents (Figure 10).

In contrast to widely used *Trichoderma* strains (e.g., *Trichoderma harzianum*), which typically rely on induced systemic resistance and limited nutrient mobilization to promote plant growth [47], strain V3 exhibits a unique combination of protease and cellulase activities along with coordinated regulation of auxin signaling through ARF genes, conferring a distinct advantage in enhancing rhizosphere nutrient availability and improving fruit nutritional quality. This study fills the research gap regarding the beneficial effects of *Chaetomium* sp. on tomatoes. This profile positions strain V3 as a promising alternative for sustainable tomato cultivation.

## 5. Conclusions

The endophytic fungus *Chaetomium* sp. strain V3 was successfully isolated from ragweed. This strain produces protease and cellulase and secretes plant hormones, including IAA, GA_3_, and zeatin. Pot and field experiments have confirmed its significant growth-promoting effects on tomato plants, demonstrating increases in plant height, root length, lateral root number, and leaf chlorophyll content, along with enhanced photosynthetic efficiency and metabolic activity. With respect to fruit yield and quality, V3 treatment increases tomato fruit weight and significantly elevates VC and lycopene contents, thereby improving fruit nutritional quality without compromising flavor or safety. Furthermore, V3 modulates the expression of tomato ARFs, including root-specific ARF2, ARF7, ARF8, and ARF10, as well as leaf-specific ARF12, indicating its role in regulating the auxin signaling pathway. The growth-promoting and quality-enhancing effects of strain V3 are mediated by a synergistic mechanism: protease and cellulase activities improve rhizosphere nutrient availability and soil structure, while secreted phytohormones regulate tissue-specific ARF gene expression. This ARF-mediated regulatory network enhances root nutrient uptake capacity, stem vascular transport efficiency, and leaf photosynthetic capacity, promoting the accumulation of photosynthetic products and ultimately contributing to higher yields and improved fruit quality.

Although the efficacy of strain V3 has been demonstrated in both pot experiments and field trials, certain limitations remain. First, the long-term stability of V3 under diverse agroecological conditions—including inter-annual climate variability and heterogeneous soil properties—requires further validation through multi-year, multi-location field trials. Second, species identification based solely on ITS sequences may lack sufficient resolution for accurately distinguishing members of complex *Chaetomium* species groups, such as the C. *globosum* complex, where ITS sequence similarity exceeds 98%. Future research should therefore focus on: (1) assessing V3’s performance across environmental gradients and varying agricultural management practices; (2) integrating multi-locus genetic markers—such as tef1 and β-tubulin—to achieve precise taxonomic classification of closely related *Chaetomium* sp. lineages; (3) optimizing inoculation strategies—including dosage, timing, and carrier materials—to enhance adaptability to different soil types and tomato cultivars; and (4) investigating the molecular interactions between V3 and indigenous soil microbial communities, along with developing biofertilizer formulations that are both stable and practical for field application. These efforts are critical to consolidating V3’s role as a reliable and sustainable biostimulant in horticultural production.

## Figures and Tables

**Table 1:** The sequence of primers for qRT-PCR of S1 ARFs.

Gene	Forward Primer Sequence (5′-3′)	Reverse Primer Sequence (5′-3′)
ARF2	GCTGCTCTACGAGCTGCTG	CTGCTGCTGATGATGATGA
ARF4	ACGACGACGATGATGATGA	GATGATGATGCTGCTGCT
ARF7	TGCTGCTGATGATGATGC	ACGACGACGCTGCTGCT
ARF8	GATGATGATGCTGCTGCT	TGCTGCTGACGACGACGA
ARF10	ACGACGACGCTGCTGCT	GATGATGATGCTGCTGAT
ARF12	TGCTGCTGATGATGATGG	ACGACGACGCTGCTGCTA
UBI3	CAGCAGCAGCAGCAGCAG	GATGATGATGATGATGAT

## Data Availability

The original contributions presented in this study are included in the article. Further inquiries can be directed to the corresponding authors.
